# *The elephant in the room*: an exploratory study of hypertensive disorders of pregnancy (HDP) management in Indonesian primary care settings

**DOI:** 10.1186/s12875-020-01303-w

**Published:** 2020-11-26

**Authors:** Fitriana Murriya Ekawati, Ova Emilia, Jane Gunn, Sharon Licqurish, Phyllis Lau

**Affiliations:** 1 Department of Family and Community Medicine, Universitas Gadjah Mada, Level 1, Gedung Radioputro, Jalan Farmako Sekip Utara, Sleman, Indonesia; 2 Department of General Practice, University of Melbourne, Level 2&3, 780 Elizabeth St, Melbourne, Victoria 3000 Australia; 3 Department of Obstetrics and Gynaecology, Universitas Gadjah Mada/Sardjito Hospital, Jalan Kesehatan No 1, Yogyakarta, Indonesia; 4 School of Nursing and Midwifery, Monash University, Level 1, Chancellors Walk, Wellington Road, Clayton, 3800 Victoria Australia

**Keywords:** Qualitative methods, Maternal health, Pregnancy hypertension, Preeclampsia, Primary care practice

## Abstract

**Background:**

Indonesia has the highest maternal mortality rate in South East Asia, that a third of the mortality is caused by hypertensive disorders of pregnancy (HDP), including preeclampsia and eclampsia. Research suggests that maternal deaths from HDP are avoidable with appropriate initial management in primary care. However, little is known regarding the exact way HDP management is conducted in Indonesian primary care. This research aims to explore the way HDP management is provided, including its barriers and facilitators in Indonesian primary care settings.

**Methods:**

This research applied a practical qualitative methodology using interviews with a topic guide. It is guided by the implementation science framework of the Medical Research Council (MRC) framework and Practical Robust Implementation and Sustainability Model (PRISM) to design and evaluate complex healthcare interventions. Primary care key stakeholders from Yogyakarta province were recruited from May–December 2018. The interviews were conducted in face-to-face, telephone, and teleconference interviews. Data from the interviews were analysed thematically using a mix of inductive and deductive approaches.

**Results:**

A total of 24 participants were interviewed, consisting of four general practitioners, five midwives, three nurses, three obstetricians, a cardiologist, five policymakers and three women with a previous history of HDP. Referrals are the usual management performed for HDP women in primary care and the primary care providers’ practice is challenged by three identified themes: (i) providers’ limited confidence to perform HDP management, (ii) fragmented continuity of care, and (iii) community beliefs. Many participants also desired to have more focused guidance to improve HDP management in primary care practice.

**Conclusion:**

Even though Indonesian antenatal care and referrals are generally accessible, there are many challenges and fragmentation of HDP management. The most prominent challenge is the primary care providers’ lack of confidence in performing the management and the ‘elephant’ of an urgent need of practice guidelines in primary care that has never been appropriately described in the literature. Further development of an evidence-based primary care-focused guidance will potentially improve primary care providers’ skills to perform optimal HDP management and provide appropriate education to their patients.

## Background

Hypertensive disorders of pregnancy (HDP) are serious global health issues that cause significant maternal morbidity and mortality worldwide. The World Health Organisation (WHO) estimates that more than 800 women die every day because of pregnancy complications, and 80% of them are caused by direct maternal mortality, such as haemorrhage, preeclampsia and infection [1]. Preeclampsia itself is a severe form of HDP that is associated with more than 70,000 global maternal and 500,000 infant-neonatal deaths each year. Some women who survive preeclampsia may also experience severe complications from seizures, kidney failures, and strokes. The burden of HDP impacts many low to middle-income countries (LMICs), which have limited resources and ability to identify pregnant women at risk of HDP, and to perform proper HDP diagnosis and treatment [2].

In Indonesia, HDP is the second most common cause of maternal mortality. The HDP trends have increased from 21.5% in 2010 to around 30% in 2015 [3, 4]. The disease also contributes to more than a thousand women deaths, or about 30% of all Indonesian maternal mortality in 2015 [5, 6]. Therefore, improvement of HDP management essential to improve Indonesian maternal health status and meet the WHO millennium goal for maternal care [7].

In the Indonesian health care system, there are publicly funded clinics or *Pusat Kesehatan Masyarakat* (Puskesmas) that provide antenatal care and maternal health surveillance in Indonesian primary care. Antenatal services in Puskesmas are available nationally and regularly accessed by almost all Indonesian women [8]. Puskesmas also provides postnatal care and emergency treatment for pregnancy complications. Around a third of Indonesian Puskesmas can also provide midwife-led delivery services [8], including providing maternal health surveillance data monitored by local health offices. In the event of any maternal mortality cases, each of the cases will be audited by local health officers, who investigate causes of and any managements conducted prior to death. The results of the audit are then presented and discussed at a maternal-neonatal audit meeting at the district level [9, 10].

The Indonesian government has also initiated and implemented many public health programs to improve health care access for pregnant women. Programs such as ‘putting midwives in the village’ (*Bidan Desa*), Indonesian national insurance (*Jaminan Kesehatan Nasional* (JKN)), local government insurance (*Jaminan Kesehatan Daerah* (Jamkesda)), and integrated antenatal care (ANC) have been implemented to ensure women have adequate access to essential and low-cost antenatal care services in hospital [11–14]. There are also other health promotion initiatives such as ‘alert village’ (*Desa Siaga*) and ‘waiting house’ (*Rumah Tunggu*) to increase community awareness regarding pregnancy complications and to provide women with adequate transportation and accommodation supports in the hospitals [15].

Although essential maternal care services in primary care are convenient and accessible as above, little is known about the exact way Indonesian primary care providers clinically provide HDP management. Investigations about the clinical aspect of HDP management are very minimal, including evaluations of current Indonesian HDP practice standards, such as the local Yogyakarta referral manuals [11] or the national World Health Organisation-South East Region Office (WHO-SEARO) [16] guideline. The only medication and procedures mentioned in Indonesian literature are direct referrals to secondary care for any HDP cases and magnesium sulphate (MgSO4) injection for preeclampsia seizures [4, 17]. Other procedures conducted in primary care, including roles and practices of midwives, nurses and general practitioners in managing HDP in primary care are inadequately discussed [18, 19]. Therefore in this publication,  the proverbial of ‘the *elephant in the room’* is used to represent the need of clinical investigation and improvement of HDP management in Indonesian literature.

This paper reports an exploratory study that is part of a larger study to improve HDP management by developing a set of HDP management pathways in Indonesian primary care [20]. The exploratory study aims to explore the way HDP has been managed in primary care, to identify implementation barriers and facilitators of any existing HDP clinical standards in Indonesia, and to determine whether primary care clinicians are ready to change their practice in HDP management. These investigations are needed to provide a general description of the provider practice before interventions to improve HDP management can be developed.

## Methods

### Theoretical framework

This study applied a qualitative exploratory design informed by the Medical Research Council (MRC) [21] and Practical Robust Implementation and Sustainability Model (PRISM) [22] as theoretical frameworks. MRC is a generic framework to design and implement interventions in health care practice [21]. PRISM is a model in implementation science that emphasise domains/factors that contribute to and affect the intervention implementation (domains of intervention, recipients, external factors, implementation and sustainability) [22]. The ultimate aim of our larger study is to develop HDP management pathways for Indonesian primary care as an intervention to improve the providers’ practice in the disease management. In this case, both of the frameworks provide guidance on stages of the intervention development and factors that may influence the implementation of the intervention in primary care [21, 22]. The MRC framework [21] was used to inform aims of this study to better understand HDP management in Indonesian primary care before a further intervention was developed [20]. Then, the PRISM [22] model was used to guide groups of stakeholders (recipients involved in HDP management) participated in this study, such as GPs, nurses, midwives, women with an HDP history, obstetricians, cardiologist, and policymakers; and to inform the development of guiding questions used in the interviews.

### Study setting

Yogyakarta Province was selected as the study settings because of its representativeness of urban settings in Indonesia and the authors’ familiarity with its health system and culture. Compared to other Indonesian provinces, Yogyakarta has relatively better health care access and a good ratio of patients and health services. Yogyakarta has 121 public primary care clinics, of which 23 are equipped with Basic Emergency Obstetrics and Neonatal Care (BEmONC) or *Pelayanan Obstetric Neonatal Esensial Dasar (PONED)* facilities for baby delivery [23]. Patients in Yogyakarta do not need to travel more than five km to arrive at any Puskesmas and most of the maternal care services in Puskesmas are covered by *JKN* or Jamkesda or at a very affordable cost. However, despite this convenient access to essential maternal care, maternal mortality rates in Yogyakarta remain high. Thirty-four women died of pregnancy complications in 2017, and 14 of them were caused by heart diseases and preeclampsia [24].

### Participant recruitment

The inclusion criteria for the interview participants’ were GPs, midwives, nurses who worked in primary care settings, policymakers (including head of clinics, medical professional organisation leaders, insurance officers, and local health officers), obstetricians, cardiologists, and women with a history of HDP.

#### Clinicians and policymakers

Clinicians and policymaker participants were identified through the authors’ professional networks in Indonesia. They were invited to participate via a short messaging system (SMS) [25], emails, or telephone calls. They were also provided with plain language statements (PLS) and written consent forms in Bahasa Indonesia.

#### Women with HDP

Women with previous HDP history were recruited using snowballing techniques by the participating GPs and midwives. The GPs and midwives were provided with a guiding script to speak to their patients whether they were keen to participate in this project. Women who expressed an interest in participating in this study were then asked for their permission for the GPs/midwives to pass on their contact details to the first author. The first author then contacted the women for further interview approaches and provided them with the study PLS and consent forms in Bahasa Indonesia. They also had opportunities to ask herfurther additional information regarding this study before the interview was conducted.

### Data collection

The data were collected by the first author (FE) using semi-structured interviews with a topic guide to gain an in-depth perspective from the participants [26]. Primary care provider participants (GPs, midwives, nurses) were asked about their experiences and expectations of providing care for HDP women in primary care settings. Obstetrician and cardiologist participants were asked about their views, experiences of receiving referrals, and expectations for the future of HDP management in primary care. Policymaker participants were asked about their evaluation and expectation of HDP management and guidelines, and women with a history of HDP were asked about their experience of receiving HDP care and their expectations for the future of HDP management in primary care settings. The complete guiding questions used in the interviews were presented in Table 1.

**Table 1:** Guiding interview questions for the participants

Project theoretical frameworks	Group of participants involved in the exploratory phase	Guiding questions for the participants
Intervention	Primary care providers Obstetricians	How is HDP usually managed in primary care? What do you think about HDP management in primary care?
Recipients’ (individuals involved in intervention implementation) characteristic	Policymakers & key informants	What are any guidelines on HDP management in primary care? What are things that work well? What are things that need to be improved?
External Environment	Women with a history of HDP	How is your experience of having HDP treatment in primary care? What are things that work well?
*Implementation*		What are things that need to be improved?

The interviews were conducted privately face-to-face, telephone, or via Zoom™ teleconference based on the participants’ and the interviewer’s mutual preference. All interviews were conducted by the first author, who was a native Indonesian speaker and had previous experience of interviewing health providers in Indonesia. Permission to record the interviews were also sought from all participants.

### Interviewer

The first author (FE), who is a female academic GP from Indonesia and is currently undertaking a doctoral degree in Australia, conducted all of the interviews. She has been practicing as a general practitioner in Indonesia for a decade and has moderate experience of interviewing health practitioners. Her personal experience of receiving advanced HDP treatment in Australia motivates her to explore and improve HDP management in Indonesian primary care.

### Language validation

All interviews were conducted in Bahasa Indonesia to aid natural discussions with the participants, who were all Indonesians. Interview recordings were transcribed in Bahasa Indonesia and then were translated into English. A quarter of the translated transcripts were randomly selected for back-translation into Bahasa Indonesia by a native Indonesian speaker to ensure the translation validation.

### Data analysis

The data were analysed thematically using a mix of inductive-deductive approaches [26, 27]. Transcripts and interview notes were read in full. All of the interview transcripts and interview notes were uploaded to the NVivo software version 11–12 [28]. Any significant quotes and notes were highlighted and coded, and the codes were then grouped based on their similarities to establish themes and sub-themes based on the domains of PRISM [22] (deductive) as well as eliciting new themes (inductive). Three co-authors also validated the coding and coding tree, and results of the analysis were discussed between all project investigators. The reporting of findings in this study adheres to the Consolidated criteria for reporting qualitative research (COREQ) statement [29].

## Results

All of the recruitment and interviews in the study were conducted from 10 May–31 December 2018. A total of 25 participants were recruited from 35 approaches. One prospective participant who agreed to participate was eventually excluded when she revealed that she was pregnant, had just been diagnosed with pregnancy hypertension and had no postpartum care experience for HDP. The final 24 interview participants consisted of GPs (*n* = 4), midwives (*n* = 5), nurses (*n* = 3), policymakers, researchers and key informants (*n* = 5), obstetricians (*n* = 3), cardiologist (*n* = 1) and women with a history of HDP (*n* = 3). The participants’ characteristics were presented in Tables 2 and 3.

**Table 2:** Summary of the participant background

Background	Number
Participants’ background:	
GP	4
Midwives	5
Nurses	3
Specialists	4
Policymakers	5
Women with a history of HDP	3
Gender:	
Male	8
female	16
Age-ranges:	
20–30	2
30–40	12
40–50	10
Education:	
High school	3
Higher education	21
Income:	
Less than 2.5 million rupiah	3
2.5–5 million rupiahs	8
More than 5 million rupiah	13
Types of practice (non-patient):	
Public primary care clinic	8
Private clinic	6
Public hospital	2
Private hospital	1
Others	4
Working experience (non-patient):	
0–5 years	1
6–10 years	11
10–15 years	2
More than 15 years	7
Working sites (non-patient):	
Urban (Less than 15 km from city)	11
Rural (More than 15 km from city)	10

**Table 3:** Characteristics of each participant

Participant Number	Background	Gender	Age range	Working experience (non-patient)	Highest Education	Income	Workplace (non-patient)
1	Women with an HDP history	Female	30–40	N/A	University	≥ IDR 5 million	N/A
2	General practitioner	Female	40–50	> 15 years	University	IDR 2.5–5 million	Public clinic (Puskesmas)
3	Midwife	Female	30–40	6–10 years	University	IDR 2.5–5 million	Private clinic
4	Midwife	Female	30–40	6–10 years	University	IDR 2.5–5 million	Private clinic
5	Nurse	Male	40–50	> 15 years	University	IDR 2.5–5 million	Private clinic
6	Policymaker	Female	30–40	6–10 years	University	≥ IDR 5 million	University
7	General practitioner	Male	30–40	6–10 years	University	≥ IDR 5 million	Public clinic (Puskesmas)
8	Midwife	Female	20–30	6–10 years	University	IDR 2.5–5 million	Private clinic
9	General practitioner	Male	40–50	> 15 years	University	≥ IDR 5 million	Public Primary Care
10	Policymaker	Female	40–50	11–15 years	University	≥ IDR 5 million	Local health policy
11	Nurse	Male	30–40	6–10 years	University	IDR 2.5–5 million	University
12	General practitioner	Female	30–40	6–10 years	University	IDR 2.5–5 million	Public clinic (Puskesmas)
13	Policy maker	Female	40–50	> 15 years	University	≥ IDR 5 million	Public clinic (Puskesmas)
14	Midwife	Female	20–30	0–5 years	University	IDR 2.5–5 million	Public clinic (Puskesmas)
15	Obstetrician	Female	30–40	6–10 years	University	≥ IDR 5 million	Public Hospital
16	Nurse	Female	40–50	> 15 years	High school	IDR 2.5–5 million	Public clinic (Puskesmas)
17	Policy maker	Female	40–50	> 15 years	University	≥ IDR 5 million	Public clinic (Puskesmas)
18	Obstetrician	Male	40–50	> 15 years	University	≥ IDR 5 million	Public Hospital
19	Women with an HDP history	Female	30–40	N/A	High school	≤ IDR 2.5 million	N/A
20	Excluded due to had just been diagnosed with pregnancy hypertension and had no previous experience of having postpartum care for HDP						
21	Women with an HDP history	Female	40–50	N/A	High school	≤ IDR 2.5 million	N/A
22	Policymaker	Male	40–50	11–15 years	University	≥ IDR 5 million	National insurance officer
23	Obstetrician	Male	30–40	6–10 years	University	≥ IDR 5 million	Private clinic
24	Midwife	Female	30–40	6–10 years	University	≥ 5 million	Private clinic
25	Cardiologist	Male	30–40	6–10 years	University	≥ 5 million	Private hospital

Eighteen interviews were conducted over the telephone, four interviews were face-to-face interviews at the first author’s office, and two interviews were Zoom™ teleconference. Each of the participants had one interview only, and the length of the interviews ranged from 20 to 90 minutes. All interviews were audio-recorded and transcribed, except for one interview due to the participant’s refusal. In that case, notes were taken by the first author during the interview. The notes were also sent back to the participant for his comments or corrections. Any significant non-verbal language from the participants was also noted during the interviews in the first author’s fieldnotes.

Three overarching themes emerged from the analysis were providers’ confidence, continuity of care and community culture, that are explained below.

### Providers’ confidence

The providers’ confidence in their ability and skills to manage HDP in primary care was the most prominent theme identified in the data analysis. Almost all of the participants agreed that it was the GPs and midwives’ roles to screen pregnant women for HDP during routine antenatal care guided by antenatal care guidelines from the Indonesian Ministry of Health. Many of them believed that after identifying women with HDP, they should refer the women to the hospital. However, they were not clear about the exact way they provided HDP management during the referral. Many participants then disclossed that they had limited confidence in their own ability to screen and manage HDP; for instance, GP and nurse participants below felt less experienced of managing HDP cases than midwives-who were seen of having more antenatal care experience.

*“I think for pregnancy care, we are between confident and not confident. Often, we (as GPs) when manage labour and delivery, we felt that we are less confident and less capable compared to the midwives” (Participant 13).*

However, surprisingly, some midwife participants also expressed a lack of confidence in prescribing medicines for pregnant women but were comfortable with prescribing supplements. When they were asked about their confidence to treat women with HDP, some said that it was not within their scopes of practice. Some midwives further said that access to hospitals was convenient, and therefore, it was unnecessary to manage the HDP cases in primary care.

*“(smiling, shy expression) … we provide care for the normal pregnancy cases that need no medication or just the multivitamin, such as iron and calcium. When we meet HDP patients, why do we have to provide more treatment? We just refer HDP patients to the hospital” (Participant 4).*

Many GP participants further mentioned that there is no guideline available in their practice that is designed explicitly for HDP management. Some guidelines, such as the WHO-SEARO, only recommend referring the women and prescribing antihypertensive medication when necessary.

*“I don’t think we have any guidelines in practice for pregnancy hypertension, and if what you mean is certain guidelines from the association, we don’t have it. We only have skills updates from the obstetricians, including procedures on patient management, such as how to monitor the women” (Participant 2).*

*“The pink book (WHO-SEARO guideline) only includes routine antenatal care. In our Puskesmas we do not have other guidelines. However, we collaborate with private midwives practices for pregnancy hypertension management, to refer the women (to hospitals) and we do home visits” (Participant 9).*

GPs participants also confessed that they were also not aware of any guideline dissemination process. Some of them mentioned that they received very little training on HDP management in primary care, which was usually given selectively for certain types of Puskesmas in primary care.

For instance, some maternal trainings are available for BEmONC Puskesmas, and if the training includes updates for HDP management, it was only available for GPs and midwives working at the BEmONC Puskesmas and not for the other Puskesmas. Therefore, she expected more updates on HDP management to be made available for broader primary care practice.

*“We received the antenatal program guideline from the government, but without any training they will only be put on our desk. In my opinion, the training also needs to be improved. So far, only BEmONC Puskesmas gets more advanced training. Other doctors like me, are not invited. However, then HDP cases are not only present at BEmONC Puskesmas. If there are any updates, please teach us, and I believe that will potentially be adopted in our settings. However, if you recommend medication that is unavailable in Puskesmas, that will also make us more confused” (Participant 17).*

When the participants were asked about suggestions or expectations for the future of HDP management, most of them expected for more guidance on HDP management, such as on screening, pre-referral procedures, monitoring and long-term management in primary care.

*“I think if you are able to provide us, we would love more guidance in managing women with HDP, such as how is their dietary consultation or perhaps psychological consultation. Because emotion can increase blood pressure, right? Other recommendations may also include what we should observe when we should give the medication? I think it was 160mmHg, then how are the blood examinations and follow-up?” (Participant 2).*

All policymakers and some women with HDP history participants also confirmed the expectation for a better HDP management in primary care. A policymaker expressed her support for the study to develop interventions for improving HDP management. A woman with an HDP history used her own word in Indonesian-Javanese language of ‘*tanggap’*, which means quick, responsive and appropriate, to represent her expectation for primary care providers to appropriately manage HDP cases. This woman explained that her condition seemed dangerous, but her doctor only advised her to eat fruits and vegetables, having some rest, and refer her to the hospital. These managements and advice, according to her, might not be appropriate for women who are in an emergency.

*“So please, if there are any interventions to improve the pregnancy complications or preeclampsia management in primary care, please let us know. We will support it” (Participant 22).*

*“I would expect that the Puskesmas workers can be more “tanggap” (English: be more responsive, quick and appropriate). What I mean is I hope they understand the patients’ symptoms and ways to manage them. Pregnancy hypertension is an emergency. It is very dangerous is that women have high blood pressure. When I was pregnant, they only asked me to eat fruit, vegetables and refer me to the hospital” (Participant 19).*

### Continuity of care

This theme refers to the quality of maternal care access and referral system for HDP management in the study settings. Other than the primary care providers’ task to screen pregnant women above, almost all participants acknowledged that antenatal care was accessible and convenient for pregnant women. The routine antenatal care program in Puskesmas is fully-funded through the JKN scheme or local government insurances. Private midwife practices and ‘waiting house’ scheme were also convenient and available to support their temporary accommodation close to the hospital.

*“Treatments for the women are covered by the insurance, JKN and other insurance such as maternity insurance, Jamkesda, many; they can use them all, this means that patients should not have any financial constraint to visit Puskesmas or hospital” (Participant 15).*

*“Maternal care policy is good already. We provide free service, no fees at all (at the clinics), transportation, waiting house-even though it is not usually used, delivery supports, anything, but our maternal deaths are also still high” (Participant 13).*

Many participants also expressed that referrals to the hospital are accessible. Maternal health has become a priority policy in the district that no hospital may reject referrals from primary care for women with pregnancy complications. However, many participants expressed that continuity of care and information between Puskesmas and the hospital was lacking. After being referred, some GPs said that HDP women usually would have all of their monitoring at the hospital until delivery. Periodically, they would return to their clinics to renew monthly referral letters or postpartum checks after delivery; however, this deferral process to primary care often did not function appropriately. Some participants recounted situations where women died from HDP complications during postnatal periods after being discharged without adequate follow up or communication from the hospital to GPs and midwives in Puskesmas.

*“This is the missing link; patients (with maternal complication) have to be continuously monitored after they return from the hospital even though she is already in good condition. However, sometimes, patients feel that their treatment is finished once they deliver the baby. Notification from the hospital also came late as their family brought it and did not pass it to us, while Puskesmas has no opportunity to visit them yet”*. (*Participant 10)*.

Communication and collaboration between primary and secondary care were also lacking. Participants working in Puskesmas mentioned that it was not uncommon for patients to access private practices without a referral from Puskesmas. On the other hand, private practices also often did not communicate to the Puskesmas after identifying women with pregnancy complications and patients themselves were not able to inform their private practice visits to their Puskesmas providers. This poor collaboration and lack of integrated care pathways was a challenge for care provision and monitoring in Puskesmas, particularly for maternal data surveillance.

*“Women with high risks pregnancy (normally) will be referred to the hospital, or they will be assisted by our cadres (community health workers) if necessary. However, private patients from family doctor or private practice do not notify us. Even though it is allowed to refer the patients directly to the hospital, I expect they could come to us first for integrated antenatal care programs and data surveillance. After that, feel free to visit any other providers, we can also ask our cadres for doing home visits for further monitoring” (Participant 14).*

### Community culture

Many participants also described unique community beliefs they felt influencing the prevalence and outcomes for women with HDP. Several participants seemed resigned to the fact that pregnancy rates amongst local women were high and that local women were more likely to have more children than women in other regions. A participant even mentioned that pregnancy often occurred at a young age when they might not be ready or understood any complications during pregnancy. Another participant also added that preeclampsia prevalence might be predicted in certain months. He reasoned that the community liked to marry in ‘good months’, and then the women got their first pregnancy and delivered their babies at the same time.

*“Many women married in earlier ages, even high school students. I don’t think they already understand any complications that can happen during pregnancy” (Participant 18).*

“*Preeclampsia is sometimes ‘seasonal,’ people have a culture of ‘wedding season,’ weddings are often held during ‘good months,’ then women get pregnant at the same time. So that preeclampsia and eclampsia (during delivery) also happen at the same time. Usually, for the first pregnancy” (Participant 11).*

Some participants also mentioned that the community knowledge about pregnancy and pregnancy complications was superficial. Some midwives and obstetricians claimed that they had discussed health promotion with their patients. However, women with HDP history contradicted this view and suggested that more health promotion activities be provided in primary care.

“*Patients are unaware of their risks of pregnancy, even though we have explained a very detailed explanation at the hospital. However, many women looked so calm, seem that they still don’t understand that their (high) blood pressure is dangerous” (Participant 15).*

“*I don’t think other patients know more about pregnancy complications; I (myself) know because I read a lot. I expect more health promotion could be done in Puskesmas” (Participant 21).*

In relation to the HDP management, primary care provider participants expressed that community culture above challenged the HDP management they performed in practice. They added that many patients might not want referrals to hospitals, particularly women living in rural regions who have lower education level and economically disadvantaged. Due to family, geographical or economic reasons, many women have to ask for their family decision before agreeing to a referral. This time consuming process then often delays their arrival at the hospital and worsen their conditions. This argument was also proved by a woman's argument below.

“*We have explained that this (hypertension) is a pregnancy complication, but their decision to take referral takes a long time. The women have to ask her family member, her and her family member; then obviously, the referral takes longer.” (Participant 4).*

*“If I can choose, I would prefer to have the treatment in Puskesmas settings rather than the hospital because of its convenient location to our house” (Participant 19).*

## Discussion

Our study has identified various views, experiences and expectations from Indonesian primary care key stakeholders regarding HDP management in primary care. It has been demonstrated from the interviews that referral is the most common management performed in primary care for HDP women. Some significant barriers of HDP management in Indonesian primary care have also been identified, including the lack of practice confidence among the providers, limited available HDP practice guidelines, limited continuity of care between primary and secondary care and certain beliefs in the community.

Using the theoretical frameworks of MRC [21] and PRISM [22] regarding aims of our larger study to improve HDP management [20], results of the interviews highlight and confirm the need for focused clinical intervention for improving HDP management in Indonesian primary care. Our findings draw attention to ‘*the elephant in the room*’, which stipulates a need for HDP clinical practice improvement that is mutually agreed as essential by the participants but is inadequately discussed anywhere in the current Indonesian literature. Many public health programs and interventions, such as insurance, referral regulations, village midwives, and national antenatal care program in Indonesia, have been shown to increase the health care utilisation rates among pregnant women. However, they have not been followed up with ‘the elephant,’ i.e., the HDP practice guidelines in primary care to improve the providers’ practice in primary care [30, 31]. The low quality of HDP clinical management resulted from a lack of practice guidelines then impacts on women being referred to hospitals with inappropriate initial management [17, 32], that subsequently limits opportunities to save them from HDP complications.

Based on the PRISM domains [22], some intrinsic and extrinsic factors that may contribute to the future of HDP pathways development and implementation in primary care have also been described. There are apparent gaps in the providers’ training, confidence, and HDP management skills in primary care. Even though women had excellent access to antenatal care, there are also chances of the lack of communication and collaborative care between private and public clinics that may limit the benefits of continuity maternal care. Therefore, the providers’ limited confidence in practice can potentially be improved with HDP management pathways in primary care that enable them to upskill their practice before and after hospital referrals. Later, after the pathways have been developed, they can also be evaluated for their impacts on the providers’ practice improvements and perhaps also the providers’ confidence in managing women with HDP in primary care.

Some external factors of primary care practice that may contribute to HDP management and potential development of HDP management pathways have also been identified. The patients’ referral system seems functioning and is supported by district regulations for hospitals to always accept referrals for pregnancy complications and implementation of many maternal health programs. However, there are certain community beliefs that emerged as another barrier of HDP management, and this should be considered in the future of HDP pathways development and implementation, such as by providing strategies to educate and engage pregnant women and their family. Therefore, the pathways later can also be used as an educational tool for the patients aiming to improve their compliance with the management and improve collaborative care between Puskesmas, other health care providers, patients and their families.

Compared to previous studies on pregnancy care in Indonesia, we have uncovered perspectives of all clinicians involved in practice and explored the complexity of maternal surveillance and its influence on HDP management. It has also been highlighted in the study of the significant need for HDP management improvement using HDP management pathways that is inadequately covered in Indonesian literature. The study findings related to community culture complements results of research conducted by D’ambruoso et al.*,* confirming that disadvantaged women in rural areas experience difficulties to access appropriate care for pregnancy complications due to geographical conditions and their socioeconomic status [18, 33]. To which in some circumstances, the community culture is also believed as a significant contributor to the Indonesian high mortality rate as prolonged referral decisions made by their families indirectly impact on the women prognosis at the hospital [34]. The finding related to thetraining for GPs, midwives and nurses in primary care also complements results from other studies emphasising a need to upskill the providers' practice in maternal care [18, 33].

### Strengths and limitations

Our study design is robust and informed by implementation science frameworks. The MRC framework is flexible, generic and compatible to be amalgamated with the PRISM model to guide this study to explore the way HDP management is provided in Indonesian primary care. Due to time constraints, most of the interviews were conducted over the phone or via teleconference and may limit dynamic interaction between the interviewer and participants. However, the interviews were all conducted by the first author, who is a native Indonesian GP, and her background is able to optimise flows of the interviews and understanding of the participants’ responses. The interview data were also analysed thematically using inductive and deductive approaches to maximise values of our qualitative study [35].

Even though the interviews focus on HDP management and may not achieve data saturation due to limited sample participants, our study findings are able to represent general condictions of maternal care provided in Indonesian primary care practice. The involvement of broad key stakeholders in the interviews also increases the data generability, including views from women with an HDP history, that is able to confirm, validate or oppose perspectives from health care providers. Results of this study should also be extrapolated to other communities cautiously as our focus was in Yogyakarta, which might not represent the broader Indonesian population.

### Implications for further research

Primary care has an essential role in HDP management. Unfortunately, limited guidelines are available in Indonesian primary care to manage HDP women appropriately. Corresponding to aims of the study and theoretical frameworks of MRC and PRISM [21, 22], results of this exploratory study will be used to inform the next phase of our larger study to develop HDP management pathways in Indonesian primary care. The pathways development should consider contexts and challenges of HDP management in Indonesia in order to optimise their potential benefits in primary care practice.

## Conclusions

HDP management in Indonesian primary care is usually conducted by only referring patients to hospitals. There are, however, challenges of HDP management in practice settings, such as providers’ limited confidence, fragmented continuity of care, and certain beliefs in the community. The management can potentially be improved by developing HDP management pathways specifically designed for primary care informed by results of this study.

## Data Availability

The project investigators keep raw materials in this study, including interview transcripts and documented themes development process. The raw data will not be shared externally to maintain the participants’ confidentiality.

## Abbreviations

ANC: Antenatal care
BEmONC: Basic Emergency Obstetrics and Neonatal Care
COREQ: Consolidated criteria for reporting qualitative research
GPs: General practitioners
HDP: Hypertensive disorders of pregnancy
JKN: *Jaminan Kesehatan Nasional* (Engish: Indonesian national health insurance)
MRC: Medical Research Council
PONED: *Pelayanan Obstetri Neonatal Esensial Dasar* (English: see BEmONC)
PRISM: Practical Robust Implementation and Sustainability Model
Puskesmas: *Pusat Kesehatan Masyarakat* (English: public primary health care clinic)
